# Using non-invasive transcranial stimulation to improve motor and cognitive function in Parkinson’s disease: a systematic review and meta-analysis

**DOI:** 10.1038/s41598-017-13260-z

**Published:** 2017-11-01

**Authors:** Alicia M. Goodwill, Jarrad A. G. Lum, Ashlee M. Hendy, Makii Muthalib, Liam Johnson, Natalia Albein-Urios, Wei-Peng Teo

**Affiliations:** 1 Institute for Physical Activity and Nutrition (IPAN), Deakin University, Melbourne, VIC Australia; 2 Institute for Health and Ageing (IHA), Australian Catholic University, Melbourne, VIC Australia; 3 Cognitive Neuroscience Unit, School of Psychology, Deakin University, Geelong, VIC Australia; 4 Stroke Division, The Florey Institute of Neuroscience and Mental Health, Heidelberg, VIC Australia; 5 Institute for Sports, Exercise and Healthy Living (ISEAL), Victoria University, Melbourne, VIC Australia; 6 School of Exercise Science, Australian Catholic University, Ballarat, VIC Australia; 7 Silverline Research Services, Brisbane, QLD Australia

## Abstract

Parkinson's disease (PD) is a neurodegenerative disorder affecting motor and cognitive abilities. There is no cure for PD, therefore identifying safe therapies to alleviate symptoms remains a priority. This meta-analysis quantified the effectiveness of repetitive transcranial magnetic stimulation (rTMS) and transcranial electrical stimulation (TES) to improve motor and cognitive dysfunction in PD. PubMed, EMBASE, Web of Science, Google Scholar, Scopus, Library of Congress and Cochrane library were searched. 24 rTMS and 9 TES studies (*n* = 33) with a sham control group were included for analyses. The Physiotherapy Evidence Database and Cochrane Risk of Bias showed high quality (7.5/10) and low bias with included studies respectively. Our results showed an overall positive effect in favour of rTMS (SMD = 0.394, CI [0.106–0.683], p = 0.007) and TES (SMD = 0.611, CI [0.188–1.035], p = 0.005) compared with sham stimulation on motor function, with no significant differences detected between rTMS and TES (Q [1] = 0.69, p = 0.406). Neither rTMS nor TES improved cognition. No effects for stimulation parameters on motor or cognitive function were observed. To enhance the clinical utility of non-invasive brain stimulation (NBS), individual prescription of stimulation parameters based upon symptomology and resting excitability state should be a priority of future research.

## Introduction

Parkinson’s disease (PD) is a progressive neurodegenerative disorder characterised by cardinal motor symptoms such as rigidity, bradykinesia, postural instability and gait disturbances. While PD is considered a movement disorder, many individuals experience debilitating non-motor symptoms such as detriments in cognition, which affect prefrontalexecutive functions, memory, attention and visuospatial abilities^1,2^. The pathophysiological processes underpinning the cardinal motor symptoms of PD reflect overactivity of corticostriatal glutamatergic transmission, and increased GABA output from the striatum^3,4^. However, the physiological processes mediating cognitive changes are less understood and often overlooked by clinicians. Impairments in cognitive abilities that rely on connections between the prefrontal, motor cortex and striatum, such as executive functions, psychomotor speed, and attention are likely a consequence of dopamine deficiency^5,6^, while memory decline appears to be related to cholinergic pathways and protein aggregates, and may be less responsive to dopamine replacement therapy (DRT)^7^.

Symptom management for PD requires a multidisciplinary approach, including physical therapy, pharmacological DRT and anticholinergic medications, and in some cases, deep brain stimulation (DBS). However, these treatment options can have considerable long-term adverse effects^8^. The efficacy of dopaminergic medications may diminish over time^9^ and lead to compulsive behaviours^10^. Furthermore, long-term use of anticholinergic medications can increase the risk of dementia^11^, and in addition to surgical risks, DBS can induce mood, memory and personality disturbances^12^. On this basis, non-invasive brain stimulation (NBS) has been identified as an alternative therapy, with the potential to alleviate both the motor and cognitive symptoms and minimise risks associated with traditional treatment options.

The use of NBS, which includes repetitive transcranial magnetic stimulation (rTMS), and transcranial electric stimulation (TES) paradigms such as transcranial direct current stimulation (tDCS) and transcranial alternating current stimulation (tACS)^13^, have gained significant interest as an adjunct treatment for PD. While the mechanisms of how rTMS and TES modulate cortical excitability differ (rTMS induces direct and trans-synaptic neuronal activation^14^, while TES induces subthreshold neuronal membrane polarization^15,16^), both methods have been shown to induce long-term potentiation or depression (LTP/LTD)-like synaptic plasticity^17–20^. Given the alterations in cortico-striatal excitatory and inhibitory transmission observed in PD, these NBS techniques may serve to normalise the aberrant neurophysiology associated with PD by modulating the excitability of underlying cortical tissue.

The physiological response from NBS can be manipulated through different stimulation parameters. High-frequency rTMS (greater than 5 Hz) and anodal-tDCS typically increase cortical excitability whilst low-frequency rTMS (less than 1 Hz) and cathodal-tDCS result in the opposite effect^21,22^. Differential outcomes from NBS have also been attributed to the timing, duration, electrode placement, coil orientation, and the physiological state of the participant^23^. While manipulation of stimulation parameters offers the potential for individualising treatment, it also highlights the importance of understanding the magnitude of effect from different NBS protocols.

Previous reviews in PD have supported overall improvements in motor function^24–27^, but not other subsections of the Unified Parkinson's disease rating scale (UPDRS)^27^. Support for differential effects of TMS parameters (frequency and no. of pulses) have been highlighted in rTMS reviews^24,28^, however the optimal tDCS parameters have not been comprehensively examined. Moreover, no review has compared the efficacy of the different stimulation modes (TES and rTMS) on both motor and cognitive outcomes. This is important as TES is simple to apply, more portable and relatively inexpensive compared with rTMS, which may serve as a more clinically-viable tool for routine use in PD therapy.

This is the first review to compare the efficacy of two commonly applied NBS techniques on both motor performance (as measured by the unified PD rating scale [UPDRS III], gait and hand movement), and cognitive performance (executive functions, psychomotor speed, visuospatial abilities and memory) in people with PD. We also examine whether different stimulation parameters modulate these outcomes, and whether the optimal stimulation parameters are different for motor and cognitive symptoms.

## Results

### Demographics of studies using rTMS

#### Participant characteristics

Of the 33 included studies, 24 studies used rTMS (Supplementary Table 1). The mean age of participants in these studies was 64 ± 3.8 years. Sample sizes ranged from eight to 98 participants and 58% of the total sample was male. The average disease duration for all 24 studies was 8 ± 3.7 years and included disease severity that ranged from mild to moderate (Hoehn & Yahr scale [H&Y] 1–3). Additionally, 13 out of the 24 rTMS studies included participants that were classified as severe (H&Y > 3)^29–41^. The rTMS intervention was primarily performed ON medication^29,30,33–49^ with 5 studies applying rTMS OFF medication^31,32,50–52^.

### Outcome variables

To quantify motor function, 21 studies used a clinical scale such as UPDRS III^29–32,34–48,51,52^ and the abnormal involuntary movement scale (AIMS)^43^, 9 studies used measures of gait performance^32,33,37–39,41,44,51,52^ and 10 measured movements of the hand^32,33,37–39,44,48–51^. In regard to cognition, executive function and psychomotor speed were assessed in five studies using the trail making test (TMT) A & B^46,47^, Stroop colour word^47^, serial reaction time task (SRTT)^37,38^ and a finger-sequencing task^48^.

### Stimulation parameters

Fifteen studies administered high frequency TMS (>1 Hz; range 1–50 Hz)^32–35,37–39,41,44,46–51^ seven low (≤1 Hz, range 0.2–1 Hz)^29,30,36,42,43,45,52^ and two studies used both^31,40^. The most common site of rTMS stimulation was the primary motor cortex (M1)^31,32,36,38,39,45,48,50,52^ and supplementary motor area (SMA)^34,35,40,43,49,51^. Four studies stimulated the prefrontal cortex (PFC)^29,30,44,47^ and another four studies stimulated multiple sites^33,37,41,46^. The method of sham stimulation differed between studies, and was achieved through changing the coil orientation^29,31,33,41,43,47,51,52^, frontal lobe sham^44,50^, occipital lobe sham^30,32,42,46,48^, dual-coil sham TMS^34–36,40^ or a single-coil sham TMS method with low intensity and sound^37–39,45,49^.

### Demographics of studies using TES

#### Participant characteristics

Of the nine TES studies included, the mean age was 63 ± 6.7 years and the sample size ranged from 9 to 25 participants (54% male). The disease duration was 9 ± 3.4 years with most participants having mild-moderate (H&Y 1–3) disease severity^53–59^, while two studies included participants with mild-severe symptoms (H&Y 2–4)^60,61^. Seven studies performed the TES intervention ON medication^53,55,57–61^, and two studies were performed OFF (12 hours) medication^54,56^.

#### Outcome variables

Six studies measured motor function using UPDRS III^53,55,56,59–61^, one used the Timed Up and Go (TUG) test^57, and two assessed hand movements58,60^. For cognitive function, psychomotor speed was assessed using the SRTT^59,60^ and TMT A^55^, executive functions were measured by the TMT B^55^, Wisconsin card sorting test (WCST)^55^, Stroop test^55^ and visual attention task (VAT)^59^. Working memory was assessed through the n-back task^54^, and forward/backward digit span (F/BDS)^55^, and visuospatial abilities with the Hooper visual organisation test (HPVOT)^55^.

#### Stimulation parameters

Six studies used anodal TES^54,55,57,58,60,61^ with only one study applying bilateral stimulation^59^. The other two studies used different polarities across different sessions^53,56^. The duration of interventions for TES studies included studies with single-session study design^54,56–58^, five consecutive days^53,59,61^ and up to 2 weeks^55,60^. TES stimulation durations ranged from 10 to 20 mins except for two studies prescribing durations of 7^57^ and 25 mins^53^.

Five TES studies used 2 mA^55,57,59–61^, one used 1 mA^58^, while the other three studies used both 1 and 2 mA intensities on separate sessions^53,54,56^. Only two different stimulation sites were used amongst TES studies, which were the M1^53,58,59,61^, PFC^55,57^ or both^54,56,60^. To achieve sham stimulation, TES devices had an in-built sham function providing a short ramp-up and down effect, allowing for pseudo-stimulation.

### Methodological quality

The Physiotherapy Evidence Database (PEDro) scale (Supplementary Table 3) and Cochrane Risk of Bias tool (Supplementary Table 4) showed high internal validity (7.5/10) and a low risk of bias. The main sources of bias reported from both the PEDro and Cochrane risk tools were lack of concealed allocation, unclear randomisation/sequence generation procedures and lack of assessor blinding. While the lack of concealed allocation and assessor blinding may have potentially biased the findings of the studies, our analyses of publication bias suggest otherwise. However, it should be noted that due to the lack of rTMS and TES studies on cognitive function (Fig. 5B and D), caution is needed when interpreting the publication bias results.

**Figure 1:**
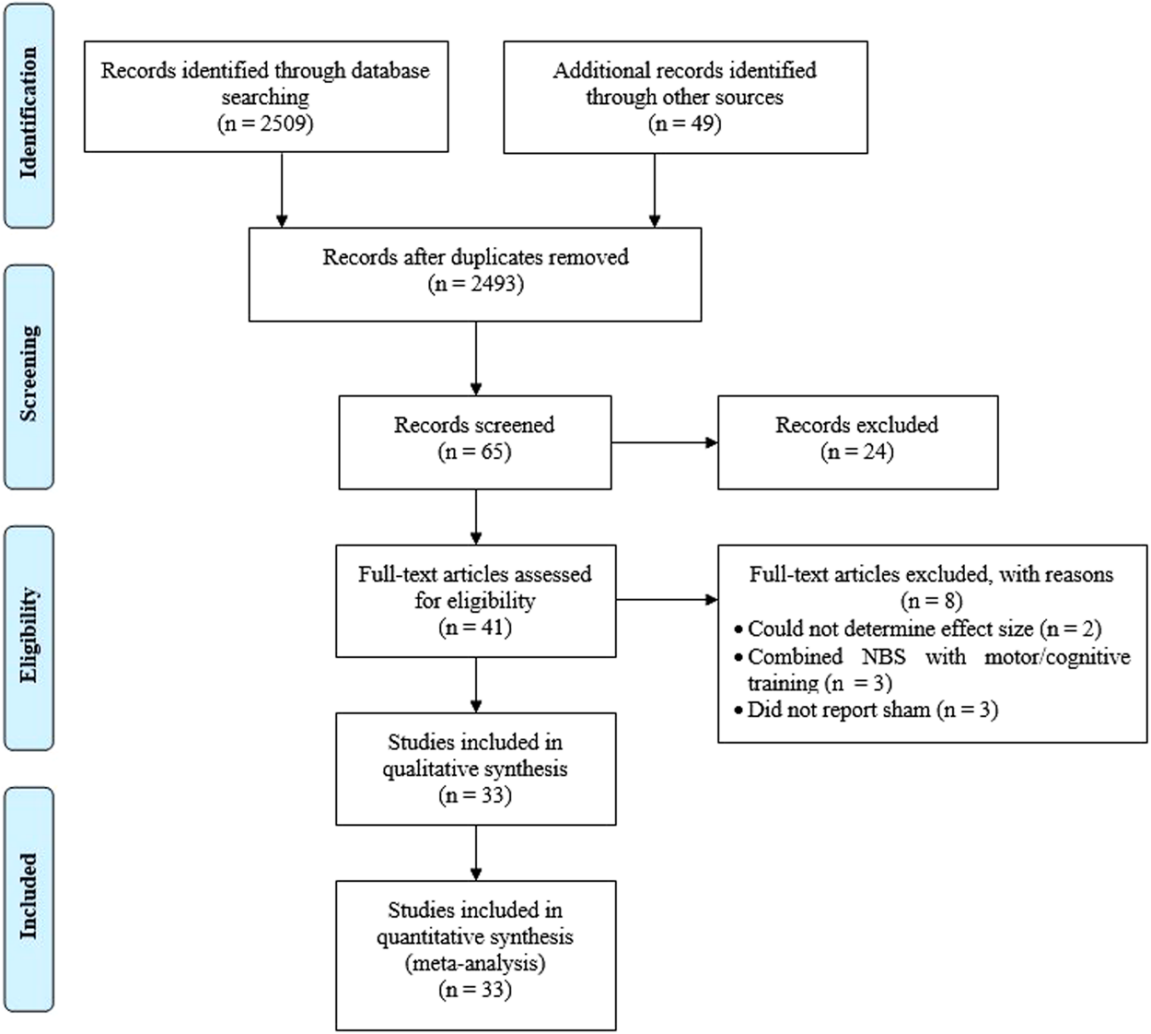
PRISMA flow diagram of study inclusion for this systematic review and meta-analysis.

### Meta-analyses

**Figure 2:**
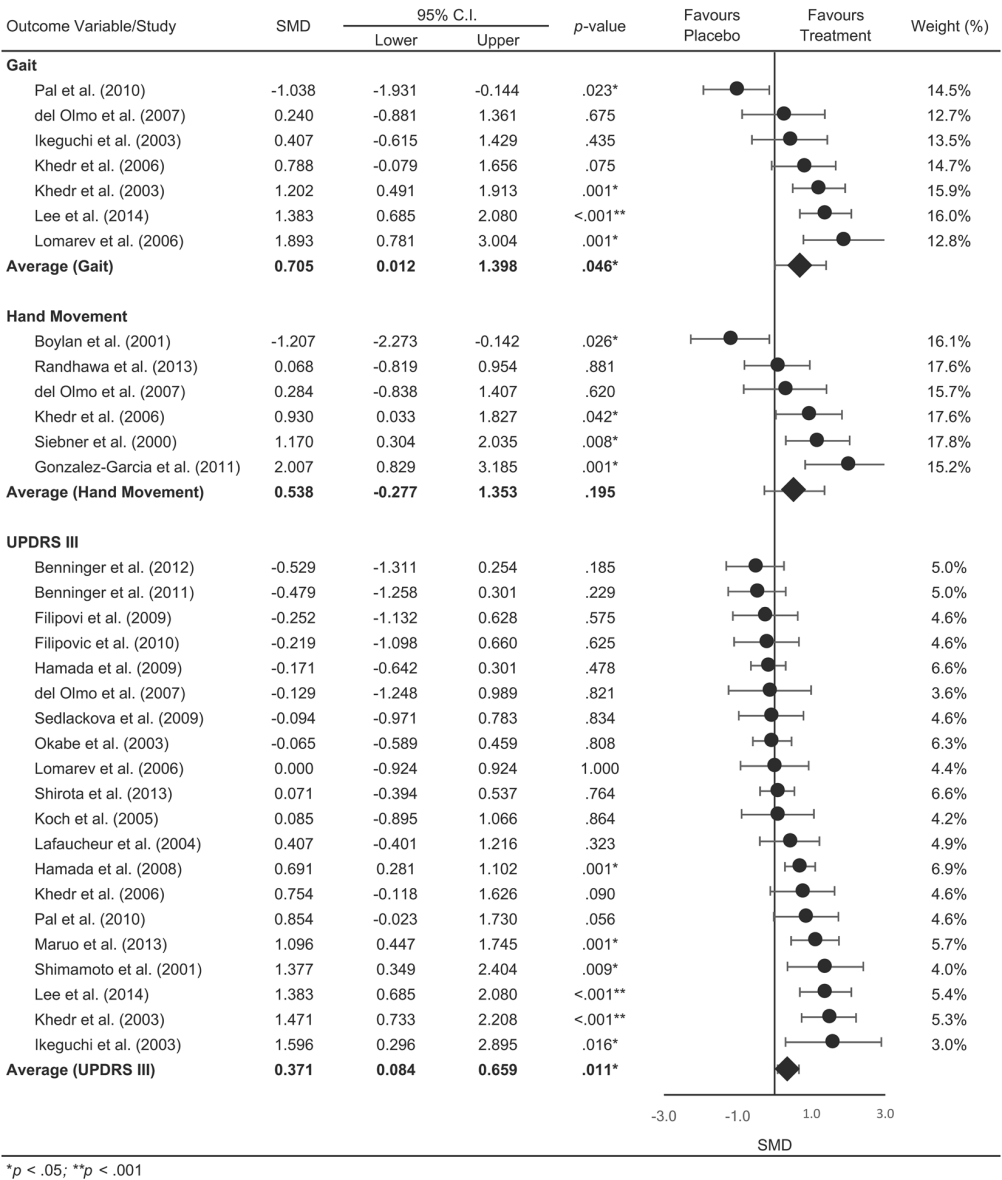
Forrest plot of studies using rTMS to improve motor function.

**Figure 3.**
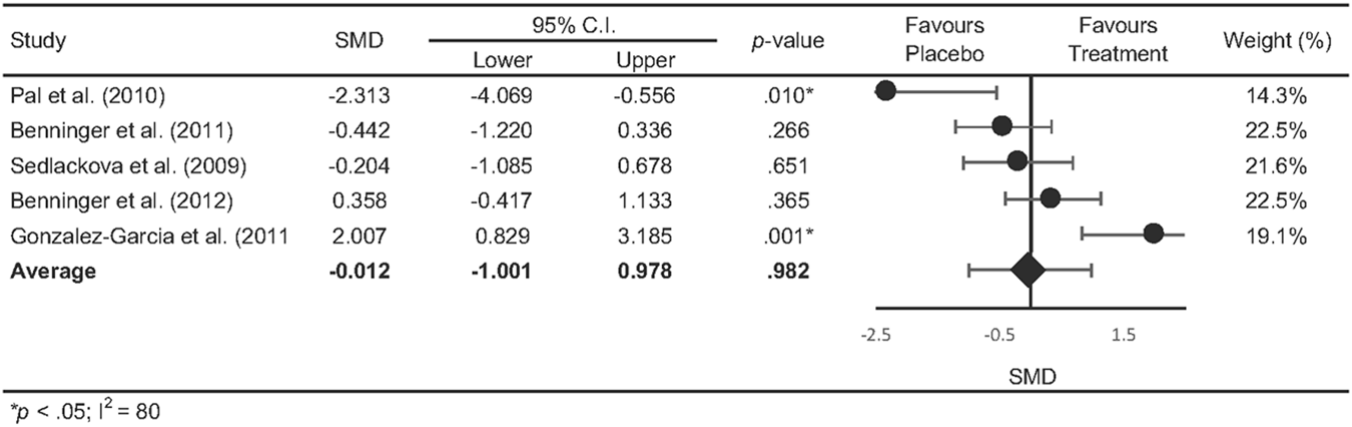
Forrest plot of overall effects of rTMS on cognitive function.

**Figure 4:**
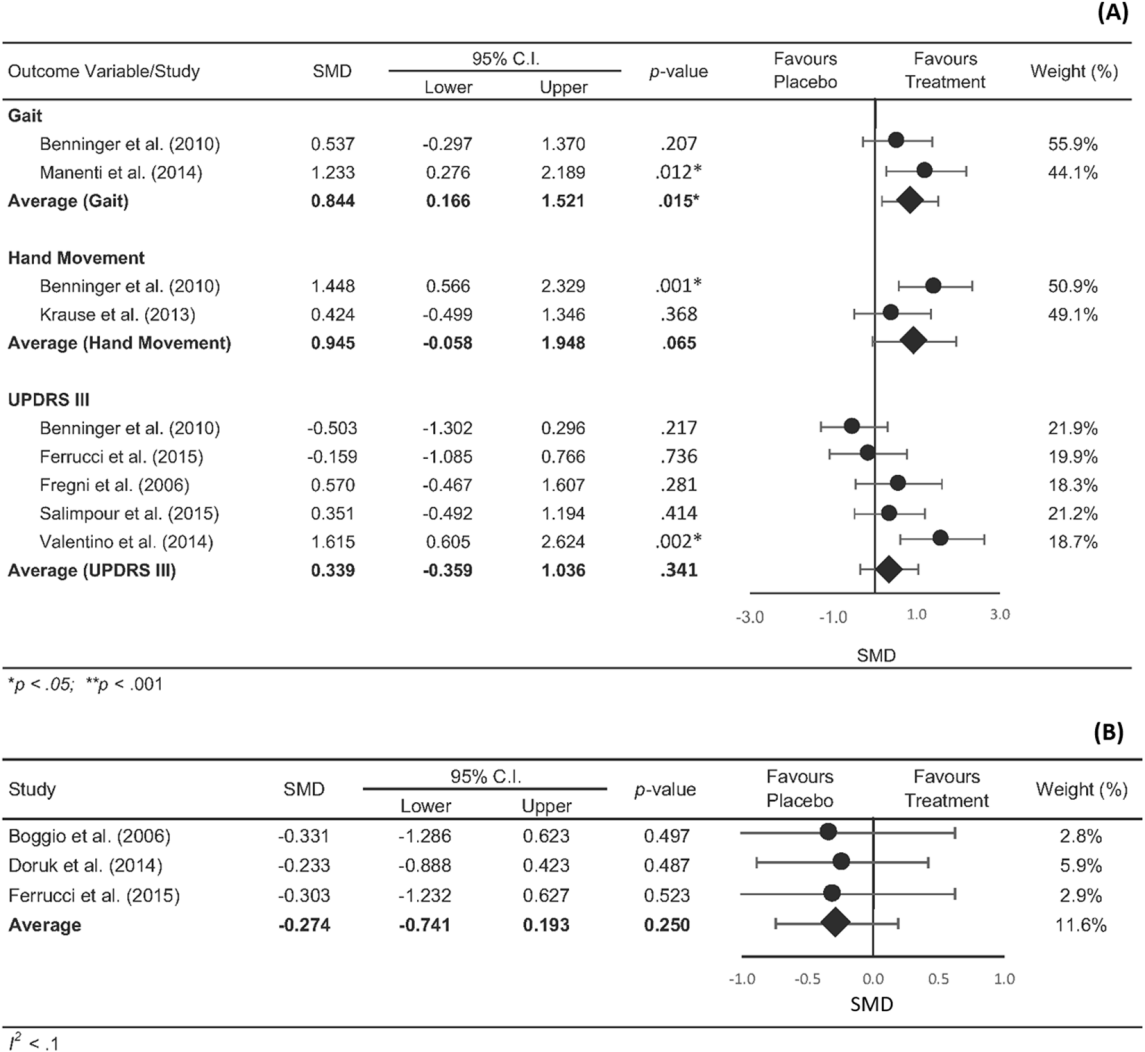
Forrest plot of overall effects of TES on (**A**) motor and (**B**) cognitive function.

**Figure 5:**
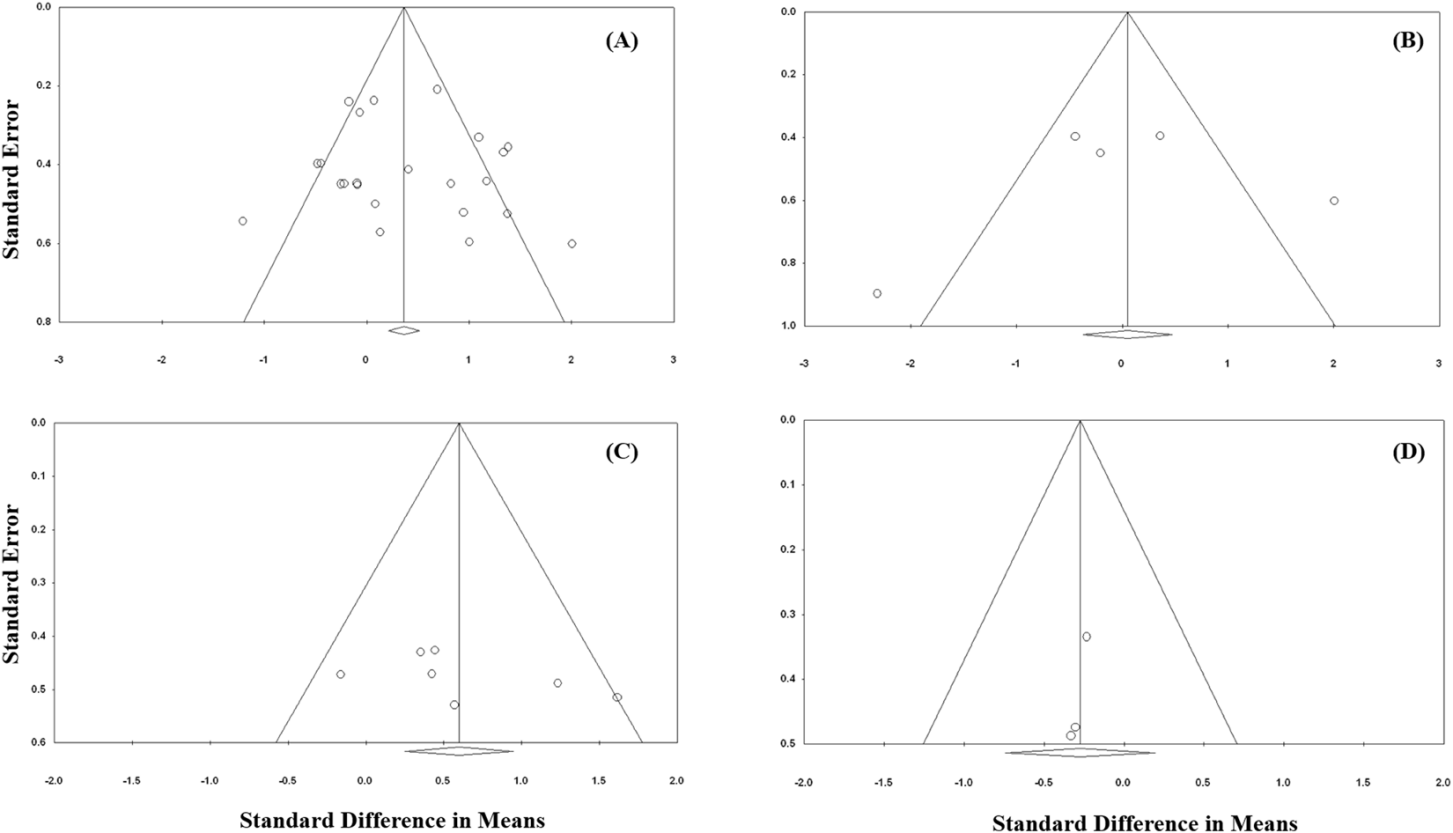
Funnel plot indicating the level of publication bias of all studies included in the meta-analysis.(**A**) rTMS motor and (**B**) rTMS cognitive function; (**C**) TES motor and (**D**) TES cognitive function.

The pooled statistics revealed an overall positive effect in favour of rTMS (SMD = 0.394, CI [0.106–0.683], p = 0.007) and TES (SMD = 0.611, CI [0.188–1.035], p = 0.005) on motor function, with no significant differences detected between rTMS and TES (Q [1] = 0.69, p = 0.406). Figure 2 shows the summary statistics for rTMS on motor outcomes separated into gait performance, hand movement and UPDRS III. The summary statistics revealed a positive effect in favour of rTMS for gait (SMD = 0.705, CI [0.012 to 1.398], p < 0.046) and UPDRS III scores (SMD = 0.371, CI [0.084 to 0.659], p = 0.011), however not for hand movements (SMD = 0.538, CI [−0.227 to 1.353], p = 0.195). Our overall summary statistic showed that rTMS did not result in significant improvements in cognitive function compared with sham stimulation (SMD = 0.271, CI [−0.43 to 0.974], p = 0.982, Fig. 3). Figure 4A shows the summary statistics for TES on motor outcome. Our results showed that TES significantly improved measures of gait (SMD = 0.844, CI [0.166 to 1.521], p = 0.015), however had no effect on hand movements although this was approaching significance (SMD = 0.945, CI [−0.058 to 1.948], p = 0.065). TES did not result in a significant improvement in overall UPDRS III scores (SMD = 0.339, CI [−0.359 to 1.036], p = 0.341). Similar to rTMS, TES did not result in significant improvements in cognitive function (SMD = −0.274, CI [−0.741 0.193], p = 0.250, Fig. 4B) compared with sham stimulation. Comparisons between rTMS and TES for measures of gait (Q [1] = 0.079, p = 0.778), hand movement (Q [1] = 0.228, p = 0.592) and UPDRS III (Q [1] = 0.030, p = 0.862) were not significantly different. The funnel plot of all included studies revealed asymmetric distribution and Egger’s tests showed no significant indication of publication bias in rTMS (motor- *t* [21] = 0.514, p = 0.613; cognition- t[3] = 0.293, p = 0.789, Fig. 5A and B) or TES studies (motor- t[5] = 1.282, p = 0.256; cognition- t[1] = 5.88, p = 0.107, Fig. 5C and D).

### Subgroup analyses

#### rTMS moderators on motor function

Subgroup mixed-effects analyses showed no significant effects of medication (SMD = 0.326 vs. 0.510; ON vs. OFF, p = 0.570), stimulation frequency (SMD = 0.433 vs. 0.363; low vs. high, p = 0.847), disease severity (SMD = 0.668 vs. 0.501 vs. 0.782; mild vs. moderate vs. severe, p = 0.876) and intensity of stimulation as a percentage of RMT (SMD = 0.336 vs. 0.519; subthreshold vs. suprathreshold, p = 0.705) and AMT (SMD = 0.147 vs. −0.245; subthreshold vs. suprathreshold, p = 0.381) on motor function outcomes. However, rTMS of the M1 (SMD: 0.692, p = 0.02) and PFC (SMD: 0.324, p = 0.04) resulted in a significant improvement in motor function compared to rTMS of the SMA (SMD: 0.253, p = 0.121) and multisite rTMS (SMD: 0.047, p = 0.321).

#### rTMS moderators on cognitive function

Mixed effects analyses did not reveal any significant effects of medication (SMD = 0.372 vs. −0.204; ON vs. OFF, p = 0.363), sites of stimulation (SMD = 0.232 vs. 0.971 vs. −0.338; M1 vs. PFC vs. multisite, p = 0.302) and stimulation intensity as a percentage of RMT (SMD = 0.214 vs. 0.125; subthreshold vs. suprathreshold, p = 0.639) and AMT (SMD = 0.333 vs. 0.159; subthreshold vs. suprathreshold, p = 0.753).

#### TES moderators on motor function

Subgroup mixed-effects analyses showed no significant effects of sites of stimulation (SMD = 0.636 vs. 0.457; M1 vs. PFC, p = 0.814), disease severity (SMD = 0.626 vs. 0.611; mild vs. moderate, p = 0.982), stimulation intensity (SMD = 0.570 vs. 0.667 vs. 0.424; 1 mA vs. 2 mA, p = 0.684) on motor function. Mixed effects analyses for TES and cognitive function was not performed due to insufficient studies in each subgroup.

#### Number of sessions to predict effect size

Meta-regression analysis showed that number of session was not a strong predictor of motor function outcomes following rTMS (R_2_ = 0.042, p = 0.298). Meta-regression of rTMS and TES on cognitive function and TES on motor function were excluded due to the low number of studies (≤5 studies) available to power the analysis.

## Discussion

This systematic review and meta-analysis included 33 studies examining the effects of rTMS and TES on motor and cognitive symptoms in individuals with PD. Building on evidence from previous meta-analyses, our study (i) provided a direct comparison between the effect sizes of rTMS and TES studies; (ii) compared the effects of rTMS and TES on both motor and cognitive function; and (iii) included a larger pool of studies than previous reviews. The results from our meta-analyses support the use of rTMS and TES to improve motor deficits in PD. Although mixed-effect analyses showed no differences in motor function between TES and rTMS, when examining specific motor domains, we showed a more widespread effect for rTMS across both UPDRS-III scores and gait parameters. However our understanding as to the benefit of NBS to improve cognitive function is limited by the smaller (5 rTMS and 3 TES) number of studies. The subgroup and meta-regression showed no differential effects of rTMS and TES moderator variables on motor or cognitive function.

Our findings suggest that rTMS of the M1 and PFC elicited the strongest effects on motor function. This agreed with a previous meta-analysis by Chou, *et al*.^24^, which showed that high-frequency (≥5 Hz) rTMS over the M1 and low-frequency (≤1 Hz) over the prefrontal areas, led to greater improvements in motor function as measured by the UPDRS III. The rationale for using high-frequency stimulation over the motor areas (i.e. M1 and SMA) appears logical, given that PD is associated with hypo-activity of these areas during self-initiated movements^62^. However, our results suggest no difference between high- and low-frequency rTMS overall, which may be in part due to large variability in results from existing studies. For example, several studies that applied 5 Hz rTMS over the SMA demonstrated significant improvements in bradykinesia^35^ and handwriting performance^49^, however Boylan, *et al*.^51^ showed that high frequency (10 Hz at 110%MT) rTMS of the SMA worsened performance in both fine and complex movements. It has been suggested that higher frequencies applied over the SMA may be counterproductive due to homeostatic plasticity^63^ caused by hyperactivity of the SMA at rest^40,64^. Of interest, our results further showed that rTMS over the PFC may improve motor function as well. While the number of studies investigating the role of the PFC in motor function in PD is limited, it is possible that stimulating the PFC increases endogenous dopamine production from the basal ganglia via corticostriatal pathways^65,66^, which may serve to alleviate motor deficits. Based on the current evidence, the effects of rTMS seem to be largely site-specific (i.e. M1, SMA or PFC) and dependent on the outcome measures to be improved. Due to the inherently large variability in rTMS responses in PD^67^, the clinical utility of rTMS might benefit from individually-tailored parameters, to more effectively manage cognitive or motor impairments in PD.

Our results from TES studies indicate a significant positive effect for measures of gait performance but not for measures of hand movement (albeit approaching significance, P = 0.065) and UPDRS III. Furthermore, our comparison between the overall pooled effects of rTMS and TES showed no significant difference in motor improvements. While the specific mechanisms by which TES improves motor function in people with PD are unclear, a proposed mechanism is that cortical stimulation may have far-reaching effects on subcortical brain structures such as the basal ganglia, via indirect cortico-subcortical projections^68^. Indeed a recent study in humans showed that the effects of TES could  extend to subcortical structures^69^ and potentially normalise dysfunctional inhibitory circuits within the basal-thalamic-cortico pathways^18,20^. Based on our findings, the fact that the TES pooled effect in motor improvements was similar to rTMS may therefore have greater clinical relevance due to its relatively inexpensive and portable nature compared with rTMS systems^70^. In this context, TES can also be applied concurrently or as a priming technique with physical therapy^71,72^. It is plausible that this application may reinforce LTP-like processes^73,74^, promoting greater retention of gains from physical therapy^75,76^. Although beyond the scope of this review, comparisons of TES applied in isolation or with physical therapy on longer-term management of motor symptoms should be a focus of future research.

In line with a previous systematic review by Broeder, *et al*.^26^, our overall pooled results suggest that TES had a greater effect on motor function and to a lesser extent on cognitive function in people with PD. This is likely to be due to the inherent nature of most neuropsychological tests used (i.e., TMT and reaction time tests) in our studies, which consists of motor and cognitive components that may be affected differently by TES. In this sense, it is difficult to discern if the improvements in cognitive test scores were indeed due to better cognitive functioning following TES, or whether TES had improved motor abilities that would result in better overall test scores. While the findings from individual studies within our search seem to indicate stronger effects on working memory using anodal tDCS at a higher stimulation intensity (2 mA)^54,56^, and over the left DLPFC, more sensitive cognitive tests are required to discriminate between the effects of TES on cognitive and motor abilities. Furthermore, cognitive processing is likely to be underpinned by several brain regions and neural networks, which makes it difficult to identify specific regions to stimulate. This is is unlike motor areas such as the M1, SMA and premotor area that exerts direct control over motor movements. It should also be noted that most TES studies included in this meta-analysis were anodal in nature, and future studies including a cathodal tDCS component will be required to determine any polarity-specific effects of TES on either motor or cognitive function in PD.

While our results demonstrated overall improvements in motor function, several limitations need to be acknowledged. Studies from both rTMS and TES demonstrated modest effect sizes (0.4–0.6) and large heterogeneity between studies. Clinical and lifestyle variables including PD-related comorbidity, physical activity levels and other mental health conditions were not accounted for in our subgroup analyses, which may have influenced the responsiveness to NBS. Other such factors that may have also contributed to heterogeneity between studies is the method of sham stimulation and individual responsiveness to NBS. Most rTMS studies used different coil orientations as a method to provide sham stimulation, however, the validity of these methods has been brought into question given the positive correlation between increasing participant sensation and unwanted cortical activation^77^. Standardisation of a consistent sham condition for rTMS studies is needed to accurately draw conclusions about the efficacy of rTMS improving motor and cognitive symptoms in PD. Furthermore, factors such as age, genetics, circadian rhythm and underlying excitability state are also considerable factors contributing to the variability between studies^78^. Another important factor to consider is that asymmetric disease dominance will undoubtedly influence PD symptomology^79,80^ and possibly the responsiveness to NBS. To account for some of these variations, an estimated sample size of more than 30 is needed to detect a reliable between-group difference^78^, however, only 12% of the studies in this review met that criteria and are likely inadequately powered to account for individual differences. Indeed, the figures presenting the results of the meta-analyses show that the confidence intervals for the average effect size is wider for the TES studies compared to rTMS. Additionally, this review included all variant forms of rTMS and TES such as theta-burst stimulation (TBS) and transcranial alternating current stimulation (tACS). Although we consider this a strength by increasing the clinical relevance of our findings, these variant forms of NBS may have modulated motor and cognitive functions through different neural mechanisms. Lastly, to allow for greater homogeneity, our between-study comparisons excluded any follow-up time points in our meta-analyses. Future reviews should aim to determine the longer-term, retention effects of rTMS and TES in PD.

In conclusion, our meta-analysis demonstrated that rTMS and TES are both viable techniques to improve motor symptoms in individuals with PD, particularly gait. Our knowledge towards cognitive improvements from NBS is limited by an insufficient number of studies and inadequate understanding of the neurobiological underpinning of cognitive impairment in PD. There was no differential effect of stimulation parameters on motor or cognitive outcomes in this review. To be able to translate NBS into a viable form of clinical treatment, a better understanding of how different NBS parameters influences motor and cognitive function is necessary to elicit optimal improvements in function in people with PD.

## Methods

### Study design and registration

Our systematic review and meta-analysis adhered to the Preferred Reporting Items for Systematic Reviews and Meta-Analysis (PRISMA) guidelines (Fig. 1) and is registered with the International Prospective Register of Systematic Reviews (PROSPERO Registration number CRD42016035699).

### Literature search strategy

Studies included in this meta-analysis were searched through PubMed, EMBASE, Web of Science, Google Scholar, Scopus, Library of Congress and Cochrane library from inception to 26^th^ February 2016, with an additional search updated through to 3^rd^ February 2017. Keywords used to search the databases include a combination of the following terms “Parkinson’s disease”, “motor function”, “cognitive function”, “non-invasive brain stimulation”, “repetitive transcranial magnetic stimulation”, “transcranial direct current stimulation”, “transcranial stimulation”, “repetitive TMS”, “transcranial DC stimulation”, “transcranial electrical stimulation”, “rTMS”, “tDCS” and “tACS”. In addition, the reference lists from existing systematic reviews and meta-analyses, and studies included in these reviews were searched to identify any additional relevant articles.

### Inclusion/exclusion criteria for study selection

Figure 5 shows the flow diagram for study inclusion/exclusion in accordance with the Preferred Reporting Items for Systematic Reviews and Meta-Analysis (PRISMA) guidelines. Articles identified from the search were exported into reference management software (EndNote X7; Thompson Scientific, New York, NY, US) and screened for duplication. Following the removal of duplicates, titles and abstracts were screened to identify relevant studies. The inclusion criteria included:

1. Study population included people with idiopathic PD;
2. Interventions using rTMS or TES;
3. Sham stimulation as a control;
4. Outcome measures for motor function including:
  - Clinical motor assessments – including UPDRS-III, H&Y, ADL, AIMS, CDRS, PPT, TUG and FOG-Q;
  - Gait kinematic as measured using a force platform, accelerometers or 3D motion capture;
  - Finger and/or wrist movement using custom-made systems such as goniometers and touchpads.
5. Validated neuropsychological tests to assess:
  - Learning and memory;
  - Executive functioning;
  - Visuospatial abilities;
  - Psychomotor speed.
6. cross-over or parallel study design;
7. Written in the English language;
8. Published in peer-reviewed scholarly journals.

The exclusion criteria included:

1. No data available to determine effect sizes,
2. No control group,
3. Case reports,
4. Studies using TES or rTMS combined with another therapy.

Studies were excluded if the title or abstract were not relevant to, or did not fit the inclusion criteria. If the title or abstract was unclear, the article was assessed in its entirety. To increase the accuracy of the screening process, the first 30% of titles and abstracts were reviewed by two assessors (WPT and AMG) with the remaining titles/abstracts screened by WPT. Two authors screened all full text articles and discrepancies were resolved via discussion. Details of all studies included in this study may be found in Supplementary Tables 1 and 2.

### Methodological quality

Two assessors (AMH and LJ) assessed the methodological quality of all studies using the Physiotherapy Evidence Database (PEDro) rating scale^81^, whilst another two assessors (MM and NAU) assessed the risk of bias of all studies using the Cochrane risk of bias assessment^82^. The PEDro scale (rated 1–10) assesses study quality in five domains; group allocation, blinding, attrition, statistical analyses and data variability. The Cochrane risk of bias tool for randomised controlled trials rates trial quality on seven domains: sequence allocation, allocation concealment, blinding, incomplete outcome data, selective outcome reporting & other sources of bias. Both pairs of assessors reviewed each study independently, and any discrepancies were graded by a third assessor (WPT). Details of the PEDro score and Cochrane risk of bias assessment may be found in Supplementary Tables 3 and 4.

### Data extraction

For all included articles, data extraction involved the retrieval of study characteristics (author, year, sample size and study design), participant demographics (age, sex, medication and disease severity), stimulation protocols (type, intensity, duration and site) and the test used to quantify motor and cognitive function outcome measures (refer to Table 1 for types of motor and cognitive tests used). Measures of mood and depressive scales (i.e. Hamilton depression scale, Beck depression scale and Geriatric depression scale) were not included in the analysis. For each included study, quantitative data for pre- and post-intervention, for both the stimulation and control (sham) conditions were extracted from the results text, tables and figures. To increase reliability, two authors (AMG & WPT) extracted the data from each study. Where studies reported more than one ‘post’ assessment, the time-point immediately following the last intervention session was extracted and used in the analysis. If studies reported multiple cognitive or motor tests, an overall weighted mean combining the effect size for each test was used to calculate a single effect size for that study. The data extracted varied depending on the reported results. This included pre-post means and standard deviations (SD; or conversion of SEM to SD) for real and sham NBS and the p-value representing the group (sham vs. real) by time (pre-post) interaction between the stimulation conditions. A plot digitising software (PlotDigitizer) was used to extract data from figures when data was not readily available from text or tables.

### Statistical analyses

As systematic influences and random error were predicted to be present between study level effect sizes, a random effects meta-analysis was performed to compare the overall pooled standardised mean differences (SMDs) for motor and cognitive function between rTMS and TES studies. Separate meta-analyses were conducted for each of the 3 categories of motor outcomes (i.e. gait, hand movements and UPDRS III) for both rTMS and TES studies and presented as averaged SMD and 95% confidence interval (CI) values. Cognitive outcomes for rTMS and TES studies were presented as an overall SMD and 95%CI value due to an insufficient number of studies to perform separate meta-analyses.

 For each study, SMD was computed such that positive values indicate that the treatment group’s performance on motor and cognitive tests was superior to sham on the outcome variable^83^. Subgroup analyses and meta-regression were agreed upon *a*-priori to assess the influence of moderator variables of rTMS on cognitive and motor function and moderator variables of TES on motor function. Subgroup and meta-regression analyses were excluded if there were insufficient studies for comparison. Where studies had more than one subgroup comparison, they were removed from the subgroup analysis^84^. These moderator variables included:

1. Medication status during intervention - ON vs. OFF medication;
2. Disease severity (Hoehn & Yahr [H&Y] score) – H&Y 1–3 (mild to moderate) vs. H&Y 2–4 (moderate) vs. H&Y 4–5 (moderate to severe);
3. Stimulation site - (primary motor cortex (M1) vs. prefrontal cortex (PFC) vs. supplementary motor area (SMA);
4. rTMS intensity (expressed as percentage of motor threshold [%MT]) – subthreshold ≤ 90% RMT/AMT) vs. suprathreshold (≥100% RMT/AMT);
5. rTMS frequency - low frequency (≤1 Hz) vs. high frequency (>1 Hz);
6. Number of stimulation sessions (continuous variable);
7. TES intensity – 1 mA vs. 2 mA;
8. TES mode – Anodal vs. tACS.

Heterogeneity was measured using the *I* ^2^ statistic, which indicates the percentage variance between studies with cut off points corresponding to low (25%), moderate (50%) and high (75%) heterogeneity^85^. Funnel plots assessed publication bias using Egger’s regression test (where non-significant asymmetry indicated no bias)^86^. All statistical analyses were performed using Comprehensive Meta-Analysis (V3.0, Biostat, Englewood, USA) using an alpha level of p < 0.05 was used to determine significance.

## Electronic supplementary material

Supplementary Tables
