# Supplementary material for: Using non-invasive transcranial stimulation to improve motor and cognitive function in Parkinson’s disease: a systematic review and meta-analysis
Source: Sci Rep. 2017 Nov 1;7:14840. doi: 10.1038/s41598-017-13260-z (PMC5665996; doi:10.1038/s41598-017-13260-z)
Supplement: Supplementary file 1 — Supplementary Tables [file 41598_2017_13260_MOESM1_ESM.pdf]

Using non-invasive transcranial stimulation to improve motor and cognitive function in Parkinson's disease: a systematic review and meta-analysis

Alicia M Goodwill,[1,2] Jarrad AG Lum,[3] Ashlee M Hendy,[1] Makii Muthalib,[3,7] Liam Johnson,[4,5,6] Natalia Albein-Urios,[3] Wei-Peng Teo[1*]

[1]Institute for Physical Activity and Nutrition (IPAN), Deakin University, Melbourne, VIC, Australia

[2]Institute for Health and Ageing (IHA), Australian Catholic University, Melbourne, VIC, Australia

[3]Cognitive Neuroscience Unit, School of Psychology, Deakin University, Geelong, VIC, Australia

[4]Stroke Division, The Florey Institute of Neuroscience and Mental Health, Heidelberg, VIC, Australia

[5]Institute for Sports, Exercise and Healthy Living (ISEAL), Victoria University, Melbourne, VIC, Australia

[6]School of Exercise Science, Australian Catholic University, Ballarat, VIC, Australia

[7]Silverline Research Services, Brisbane, QLD, Australia

CORRESPONDING AUTHOR:

Dr Wei-Peng Teo,

Institute for Physical Activity and Nutrition (IPAN),

Deakin University,

221 Burwood Highway,

Burwood, VIC, 3125, Australia.

Email: Weipeng.teo@deakin.edu.au

Supplementary Table 1. Study characteristics of included studies for both rTMS and TES

Reference	N (% male)	Age (mean yrs)	Disease duration (mean yrs)	H&Y stage	Medication during intervention	Outcome measure (Motor/Cognitive)
**rTMS**						
**Siebner et al. (2000)**	12 (33)	58	6.3	1-2.5	Off	Motor: Finger ballistic aiming task
**Boylan et al. (2001)**	10 (60)	63.5	6.6	2.0-3.0	Off	Motor: UPDRS III, Hand RT and MT, TUG, timed HPS
**Shimamoto et al. (2001)**	18	65	7	1.5-4.0	On & Off	Motor: H&Y scale, England ADL scale, UPDRS III
**Okabe et al. (2002)**	85 (60)	67	8.5	2.0-3.0	On	Motor: Total and UPDRS III
**Ikeguchi et al. (2003)**	12 (25)	69	7.8	1.0-4.0	On	Motor: ADL, UPDRS III
**Khedr et al. (2003)**	36 (69)	57.7	3.26	2.0-3.0	Off	Motor: UPDRS III, Walking speed
**Lefaucheur et al. (2004)**	12 (58)	64	11	2.5-4.0	On & Off	Motor: UPDRS III, Ballistic finger task, PPT
**Koch et al. (2005)**	8 (50)	60.8	16.5	-	On	Motor: AIMS, UPDRS
**Khedr et al. (2006)**						
Group 1	10	53.5	1.45	1.0-2.5	Off	Motor: UPDRS III, WT, FTT, motor self-assessment scale
Group 2	25	62.5	5.8	3.0-5.0	Off	Motor: UPDRS III, WT, FTT, motor self-assessment scale

Group 3	10	60.2	4.6	3.0-5.0	Off	Motor: UPDRS III, WT, FTT, motor self-assessment scale
Group 4	10	60.6	4.6	3.0-5.0	Off	Motor: UPDRS III, WT, FTT, motor self-assessment scale
**Lomarev et al. (2006)**	18 (83)	64.5	12.3	2.0-4.0	On	Motor: 10m WT, complex movement test
**del Olmo et al. (2007)**	13 (46)	61.7	3.8	1.0-3.0	On	Motor: UPDRS III, FTT, reaching and grip movement, gait speed
**Hamada et al. (2008)**	98 (55)	66.4	8	2.0-4.0	On	Motor: Total and UPDRS III
**Filipovic et al. (2009)**	10 (50)	64.5	15.6	-	On	Motor: UPDRS III, GCRS
**Hamada et al. (2009)**	98 (55)	66.4	8	2.0-4.0	On	Motor: UPDRS III
**Sedlackova et al. (2009)**	10 (90)	63.7	7.8	-	On	Motor: UPDRS III; Cognition: CRT, VFT, WM, TMT A&B, F/BDS
**Filipovic et al. (2010)**	10 (50)	64.5	15.6	2.0-4.0	On	Motor: UPDRS III
**Pal et al. (2010)**	22 (50)	68.5	-	2.0-3.0	On	Motor: UPDRS III, H&Y scale; Cognitive: TMT A&B, Stroop test
**Benninger et al. (2011)**	26 (69)	63.9	8.7	2.0-4.0	On	Motor: 10m WT, hands opening/closing and elbow flexion, UPDRS III; Cognitive: SRTT

**Gonzalez-Garcia et al. (2011)**	17 (65)	64.4	-	2.0-3.0	On	Motor: UPDRS III, FTT; Cognitive: Random finger sequence test
**Benninger et al. (2012)**	26 (77)	64.1	9	2.0-4.0	On	Motor: 10m WT, hands opening/closing and elbow flexion, UPDRS III, FOG-Q; Cognitive: SRTT
**Maruo et al. (2013)**	21 (47)	63	12	2.0-4.0	On	UPDRS III/10m WT/finger tapping test/self-assessment motor score/VAS of motor symptoms
**Shirota et al. (2013)**						
Group 1- 1Hz TMS	34 (41)	68.8	8.5	2.0-4.0	On	Motor: UPDRS III
Group 2- 10Hz TMS	34 (35)	67.9	7.8	2.0-4.0	On	Motor: UPDRS III
Group 3- Sham	34 (56)	65.7	7.6	2.0-4.0	On	Motor: UPDRS III
**Randhawa et al. (2013)**	10 (90)	70.5	5.1	1-1.5	On	Motor: Handwriting kinematics
**Lee et al. (2014)**	20 (70)	71.6	4.7	3.0-4.0	On	Motor: UPDRS III, turning steps and time, TUG, FOG-Q
**TES**						
**Boggio et al. (2006)**						
Exp 1- 1mA	18 (67)	59.2 ±9.9	13.7	2.3 ± 0.9	Off	n-back task
Exp 2- 2mA		61.0 ± 21.1	12.7	2.4 ± 0.7	Off	n-back task
**Fregni et al. (2006)**						
Exp 1- Anodal tDCS	9 (56)	59.2 ± 3.3	13.7	2.4 ± 0.4	Off	Motor: UPDRS III/SRT/PPT

Exp 2- Cathodal tDCS	8 (88)	65.9 ± 4.6	10.7	2.3 ± 0.3	Off	Motor: UPDRS III/SRT/PPT
**Benninger et al. (2010)**	25 (64)	63.9 ± 8.7	9.9	2.0-4.0	On	Motor: 10m WT, hands opening/closing and elbow flexion, UPDRS III; Cognitive: SRTT
**Krause et al. (2013)**	10 (50)	49.4 ± 3.1	1.9	1.0-2.0	On	Motor: Fast FFT, diadochokinesia test, isometric power test
**Doruk et al. (2014)**	18 (33)	61.0 ± 8.0	-	1.3 ± 1.1	On	Motor: UPDRS III, SRT, FT, PPT, buttoning up, HPS, 4-CRT; Cognitive: TMT A&B, WCST, PCL, F/BDS, Stroop test, HPVOT, CPM
**Manenti et al. (2014)**	10 (60)	67 1 ± 7.2	8.1	2.0-4.0	On	Motor: TUG
**Valentino et al. (2014)**	10 (50)	72.3 ± 3.6	11	2.0-3.0	On	Motor: UPDRS III, SWST, FOG-Q
**Ferrucci et al. (2015)**	9 (56)	74.3 ± 7.5	10.8	1.0-2.5	On	Motor: UPDRS III and IV; Cognitive: VAT, SRTT
**Salimpour et al. (2015)**						
Exp 1	15 (60)	60 ± 7.4	8	1.0-2.5	On	Motor: UPDRS III
Exp 2	10 (60)	60 ± 6.9	7	1.0-2.5	On	Motor: UPDRS III
Exp 3	10 (90)	62 ± 10.8	9	1.0-2.5	On	Motor: UPDRS III
Exp 4	10 (60)	61 ± 9.2	8	1.0-2.5	On	Motor: UPDRS III

ADL- Activity of daily living; AIMS- Abnormal Involuntary Movement Scale; CDRS- Clinical Dyskinesia Rating Scale; CPM- Coloured progressive Matrices; CRT- Choice reaction time; F/BDS- Forwards/backwards digit span; FOG-Q- Freezing of gait questionnaire; FTT- Finger tapping task; GCRS- Graded Clinical Rating Scale; H&Y- Hoehn & Yahr; HPS- Hand pronation-supination; HPVOT- Hooper visual organisation test; MT- Movement time; PPT- Purdue pegboard test; RT- Reaction time; SRT- Simple reaction time; SRTT- Serial reaction time test; SWST- Stand walk sit test; TMT- Trail-making test; TUG- Timed up and go; VAS- Visual analogue scale; VAT- Visual attention task; VFT- Verbal fluency task; WCST- Wisconsin card sorting test; WM- Working memory; WT- Walking test.

Supplementary Table 2. Stimulation parameters used for rTMS and TES studies

Reference	Site/coil	Freq.	Intensity	No. pulses	No. sessions
**rTMS**					
**Siebner et al. (2000)**	M1/Figure-8	5 Hz	90% rMT	2250	Single
**Boylan et al. (2001)**	SMA/Figure-8	10 Hz	110% rMT	2000	Single
**Shimamoto et al. (2001)**	Frontal (F3)/circular	0.2 Hz	700 V	30 each hemisphere	1 session/ week x 8 weeks
**Okabe et al. (2002)**	1. M1<br>2. Occipital (inion) /circular	0.2 Hz	110% aMT	100	1 session/ week x 8 weeks
**Ikeguchi et al. (2003)**	1. PFC and OCC /circular	0.2 Hz	70% max SO	30 each hemisphere	6 sessions /week x 2 weeks
**Khedr et al. (2003)**	M1/Figure-8	5 Hz	120% rMT	100 each hemisphere	10 consecutive days
**Lafaucheur et al. (2004)**					
Intervention 1 (L-dopa)	M1/Figure-8	NA	NA	NA	Single session
Intervention 2	M1/Figure-8	5 Hz	80% rMT	600	Single session
Intervention 3	M1/Figure-8	10 Hz	80% rMT	2000	Single session

**Koch et al. (2005)**					
Intervention 1	SMA/Figure-8	1 Hz	90% aMT	900	Single session
Intervention 2	SMA /Figure-8	5 Hz	110% aMT	900	
**Khedr et al. (2006)**					
Group 1	M1	25 Hz	100% rMT	1000 each hemisphere	Single session
Group 2	M1	25 Hz	100% rMT		
Group 3	M1	10 Hz	100% rMT		
Group 4	OCC/Figure-8	25 Hz	100% rMT		
**Lomarev et al. (2006)**	M1 & DLPFC/solid core	25 Hz	100% rMT	300 per site and side	2 sessions/week x 4 weeks
**del Olmo et al. (2007)**	DLPFC/Figure-8	10 Hz	90% rMT	450 (3 x 150-pulse trains)	10 consecutive days
**Hamada et al. (2008)**	SMA/Figure-8	5 Hz	110% aMT	1000 (20 x 50-pulse trains)	Single session
**Sedlackova et al. (2009)**	PMd, DLPC & OCC /Figure-8	10 Hz	100% rMT	1350 (15 x 30-pulse trains)	Single session

**Filipovic et al. (2009)**	M1/figure-8	1 Hz	90 rMT	1800 (3 x 600-pulse trains)	4 consecutive days/week x 2 weeks
**Filipovic et al. (2010)**	M1/Figure-8	1 Hz	90% aMT	1800 (3 x 600-pulse trains)	Single session
**Hamanda et al. (2009)**	SMA/Figure-8	5 Hz	110% aMT	1000 (20 x 50-pulse trains)	1 session/week x 8 weeks
**Pal et al. (2010)**	DLPC/Figure-8	5 Hz	90% rMT	600 (12 x 10-second trains)	10 consecutive days
**Benninger et al. (2011)**	M1 & DLPC	50 Hz	80% aMT	NA	4 consecutive days/week x 2 weeks
**Gonzalez-Garcia et al. (2011)**	M1/Figure-8	25 Hz	80% rMT	1000 (10 x 100-pulse trains)	5 consecutive days/month x 3 months
**Benninger et al. (2012)**	M1/Figure-8	50 Hz	80% aMT	Both hemispheres	4 consecutive days/week x 2 weeks
**Maruo et al. (2013)**	M1/Figure-8	10 Hz	100% rMT	1000	3 consecutive days

**Shirota et al. (2013)**					
Exp 1.	SMA/Figure-8	1 Hz	110% aMT	1000 (20 x 50-pulse trains)	Single session
Exp 2.	SMA/Figure-8	10 Hz		1000 (20 x 50-pulse trains)	
**Randhawa et al. (2013)**	SMA/Figure-8	5 Hz	110% of rMT	1200	Single session
**Lee et al. (2014)**	M1 & SMA/Double cone<br>DLPFC/Figure-8	10 Hz	90% rMT	1000 (20 x 50-pulse trains)	Single session

Reference	Site	Electrode montage	Intensity/electrode surface/<br>current density	Duration	No sessions
**TES**					
**Boggio et al. (2006)**					
Exp 1	Left DLPC and M1	Anodal	1mA/35cm^2/0.029mA.cm^2	20 mins	Single session
Exp 2	Left DLPC and M1		2mA/35 cm^2/0.057mA.cm^2		
**Fregni et al. (2006)**					
Exp A	M1	Anodal	1mA/35cm^2/0.029 mA.cm^2		
Exp B	M1	Cathodal	1mA/35cm^2/0.029mA.cm^2	10 mins	Single session

Exp C	DLPFC	Anodal	1mA/35cm^2/0.029mA.cm^2		
**Beninger et al. (2010)**	M1 and PFC	Anodal	2mA/97.5cm^2/0.021 mA.cm^2	20 mins	3/week x 2.5 weeks (8 total)
**Krause et al. (2013)**	M1	Anodal (tACS)	1mA/35cm^2/0.029mA.cm^2	15 mins	Single session
**Doruk et al. (2014)** Group 1.	Left DLPFC	Anodal	2mA/35cm^2/0.057mA.cm^2	20 mins	5 consecutive/week x 2 weeks
Group 2.	Right DLPFC				
**Manenti et al. (2014)**	Left or right DLPFC	Anodal	2mA/35cm^2/0.057mA.cm^2	7 mins	Single session
**Valentino et al. (2014)**	M1	Anodal	2 mA	20 mins	1/day x 5 consecutive days
**Ferrucci et al. (2015)**	M1	Bilateral	2mA/35cm^2/0.057mA.cm^2	20 mins	1/day x 5 consecutive days
**Salimpour et al. (2015)**					

Exp 1.	M1	Bilateral	1mA/25cm^2/0.04mA.cm^2		Single session
Exp 2.	M1	Bilateral	2mA/25 cm^2/0.08mA.cm^2	25 mins	Single session
Exp 3. Double blinded tDCS	M1	Anodal or cathodal	2mA/25 cm^2/0.08mA.cm^2		3 consecutive days
Exp 4. Long term tDCS	M1	Bilateral	2mA/25cm^2/0.08mA.cm^2		5 consecutive days

aMT- Active motor threshold; DLPFC- Dorsolateral prefrontal cortex; M1- Primary motor cortex; PMd- Dorsal premotor cortex; SMA- Supplementary motor area; OCC- Occipital cortex; rMT- Resting motor threshold; tACS- Transcranial alternating current stimulation.

Supplementary Table 3. Quality of study design as measured by the PEDro scale.

CRITERIA	2	3	4	5	6	7	8	9	10	11	(/10)
**rTMS studies**											
Siebner et al. (2000)	Y	NA	Y	Y	N	N	Y	Y	Y	Y	7
Boylan et al. (2001)	Y	Y	Y	Y	N	N	Y	Y	Y	Y	8
Shimamoto et al. (2001)	N	N	Y	Y	N	N	Y	Y	N	Y	5
Okabe et al. (2002)	Y	N	Y	Y	N	Y	N	Y	Y	Y	7
Ikeguchi et al. (2003)	N	N	Y	N	N	N	Y	Y	Y	Y	6
Khedr et al. (2003)	Y	Y	Y	Y	N	Y	Y	Y	N	Y	8
Lafaucheur et al. (2004)	Y	NA	Y	Y	N	Y	Y	Y	Y	Y	8
Koch et al. (2005)	N	NA	Y	Y	N	Y	Y	Y	Y	Y	7
Khedr et al. (2006)	N	N	N	Y	N	N	Y	Y	Y	Y	5
Lomarev et al. (2006)	Y	Y	N	Y	N	Y	Y	Y	Y	Y	8
del Olmo et al. (2007)	Y	Y	Y	Y	N	N	Y	Y	Y	Y	8
Hamada et al. (2008)	Y	Y	Y	Y	N	Y	Y	Y	Y	Y	9
Sedlackova et al. (2009)	Y	NA	Y	Y	N	N	Y	Y	Y	Y	7
Filipovic et al. (2009)	Y	NA	Y	Y	N	N	Y	Y	Y	Y	7
Hamada et al. (2009)	Y	Y	Y	Y	N	Y	Y	Y	Y	Y	9
Filipovic et al. (2010)	Y	NA	Y	Y	N	N	Y	Y	Y	Y	7
Pal et al. (2010)	Y	Y	Y	Y	N	Y	Y	Y	Y	Y	9
Benninger et al. (2011)	Y	Y	N	Y	Y	Y	Y	Y	Y	Y	9
Gonzalez-Garcia et al. (2011)	N	N	Y	N	N	N	Y	Y	Y	Y	5
Benninger et al. (2012)	Y	Y	Y	Y	Y	Y	Y	Y	Y	Y	10
Maruo et al. (2013)	Y	NA	Y	Y	N	Y	Y	Y	Y	Y	9
Shirota et al. (2013)	Y	NA	Y	Y	N	Y	Y	Y	Y	Y	8
Randhawa et al. (2013)	Y	NA	Y	Y	Y	N	Y	Y	Y	Y	8
Lee et al. (2014)	Y	NA	Y	Y	N	Y	Y	Y	Y	Y	8
**TES studies**											
Boggio et al. (2006)	Y	N	Y	Y	N	N	Y	Y	Y	Y	7
Fregni et al. (2006)	N	N	Y	Y	N	Y	Y	y	y	y	7
Benninger et al. (2010)	Y	N	Y	Y	N	Y	Y	Y	Y	Y	8
Krause et al. (2013)	Y	N	Y	Y	N	Y	Y	Y	Y	Y	8
Doruk et al. (2014)	Y	N	Y	Y	N	Y	N	Y	Y	Y	7
Manenti et al. (2014)	N	N	Y	Y	N	Y	Y	Y	Y	Y	7
Valentino et al. (2014)	Y	N	Y	Y	N	Y	Y	Y	Y	Y	8
Ferrucci et al. (2015)	Y	N	Y	Y	N	Y	Y	Y	Y	Y	8
Salimpour et al. (2015)	N	N	N	Y	N	N	N	Y	Y	Y	4
										Mean =	7.5

Supplementary Table 4. Assessment of bias using the Cochrane risk of bias assessment scale.

	Sequence generation	Allocation concealment	Blinding of participants, personnel and outcomes	Incomplete outcome data	Selective outcome reporting	Other source of bias
rTMS studies						
Siebner et al. (2000)	L	U	L	L	U	U
Boylan et al. (2001)	L	U	L	L	L	U
Shimamoto et al. (2001)	U	U	U	L	L	L
Okabe et al. (2002)	L	U	L	L	L	L
Ikeguchi et al. (2003)	U	U	U	L	L	U
Khedr et al. (2003)	L	L	L	L	L	L
Lafaucheur et al. (2004)	U	U	L	L	L	L
Koch et al. (2005)	U	U	U	L	L	L
Khedr et al. (2006)	U	U	U	L	L	L
Lomarev et al. (2006)	U	U	U	L	L	L
del Olmo et al. (2007)	L	U	U	L	L	L
Hamada et al. (2008)	L	U	L	L	L	L
Filipovic et al. (2009)	L	U	L	U	U	L
Hamada et al. (2009)	L	L	L	L	L	L
Sedlackova et al. (2009)	U	U	U	L	L	L
Filipovic et al. (2010)	L	U	L	U	U	L
Pal et al. (2010)	L	L	L	L	L	L
Benninger et al. (2011)	L	L	U	L	L	L
Gonzalez-Garcia et al. (2011)	U	U	L	L	L	L
Benninger et al. (2012)	L	L	L	L	L	L
Maruo et al. (2013)	L	L	L	L	L	L
Shirota et al. (2013)	L	L	L	L	L	L
Randhawa et al. (2013)	U	L	L	L	L	U
Lee et al. (2014)	L	U	U	L	L	L
TES studies						
Boggio et al. (2006)	U	U	U	L	L	L
Fregni et al. (2006)	U	U	L	L	L	U
Benninger et al. (2010)	L	L	L	L	L	L
Krause et al. (2013)	U	U	L	L	U	L
Doruk et al. (2014)	L	L	L	L	L	L
Manenti et al. (2014)	U	U	U	L	L	L
Valentino et al. (2014)	L	U	L	L	L	L
Ferrucci et al. (2015)	L	U	L	L	L	L
Salimpour et al. (2015)	U	U	U	L	L	L
Low risk	19	10	21	31	29	28
High risk	0	0	0	0	0	0
Unclear	14	21	12	2	3	3
